# New record of the genus *Manipuria* Jacoby (Chrysomelidae, Criocerinae) from China, with description of a new species

**DOI:** 10.3897/zookeys.1009.59693

**Published:** 2021-01-04

**Authors:** Yuan Xu, Wenxuan Bi, Hongbin Liang

**Affiliations:** 1 College of Life Sciences, Anhui Normal University, Anhui 241000, China Anhui Normal University Anhui China; 2 Room 401, No. 2, Lane 155, Lianhua South Road, Shanghai, 201100, China Institute of Zoology, Chinese Academy of Sciences Beijing China; 3 Key Laboratory of Zoological Systematics and Evolution, Institute of Zoology, Chinese Academy of Sciences, Beijing 100101, China Unaffiliated Shanghai China

**Keywords:** Coleoptera, Crioceris, Smilacaceae, taxonomy, Tibet

## Abstract

After a century since the erection of the genus *Manipuria* from India, its type species *M. dohertyi* Jacoby was discovered in Yunnan Province of China. A new *Manipuria* species, *M. yuae***sp. nov.**, is described from Tibet and Yunnan, China. The new species differs from *M. dohertyi* by its larger size, unicolored elytra, and absence of a tooth-like prolongation in front of the mandible. Additional data is provided for *M. dohertyi* based on new material from China.

## Introduction

The monotypic genus *Manipuria* Jacoby was erected in 1908 based on *Manipuria dohertyi* Jacoby, 1908 from Manipur, India. This particular species is characterized by an elongate and subcylindrical body; the head longer than wide, not strongly constricted posteriorly, with a tooth-like prolongation on the under surface in front of the mandible (Jacoby 1908). For more than a century, no other species of the genus have been found, and no other distribution records are known except for the type locality, Manipur.

During an expedition to southeastern Tibet in August 2011, three large specimens of Criocerinae were discovered on a host plant belonging to Smilacaceae. Based on the keys of Jacoby (1908) and Monrós (1960), these specimens were readily keyed out as members of the genus *Manipuria*. In the insect collection of Kunming Institute of Zoology, Kunming, China, we found another specimen of *Manipuria* collected from Ruili City of Yunnan Province. Subsequent morphological comparisons of the specimens with the type of *M. dohertyi* have convinced us that they represent another *Manipuria* species previously unknown to science. A description of this new species follows.

In August 2019, we made an expedition to Tibet and Yunnan to survey Criocerinae. When walking on a road in Maku village of Dulongjiang township, we saw a “*Lilioceris*” beetle flying quickly in front of us and then resting on a plant. We caught it with an insect net and recognized it as *M. dohertyi* from four ocular patches on the elytra. In fact, Dulongjiang represented a new distribution area, because the second author (BWX) caught this species in July 2015 from Qinlangdang, another village of Dulongjiang ca 10 km south of Maku.

The purpose of this paper is to describe the new species discovered in Tibet and Yunnan, and to provide additional data on *M. dohertyi* based on our new material from Yunnan.

## Materials and methods

This study is based on the examination of the type specimen and those collected from Tibet and Yunnan, China. The methods of specimen observation and preparation followed previous publications (Tishechkin et al. 2011; Li et al. 2013; Shi and Liang 2015). Morphological terminology follows Chou et al. (1993), Matsumura et al. (2013) and Li and Liang (2018).

Body length (**BL**) was measured from the anterior margin of labrum to the apex of elytra; body width (**BW**) was measured along the greatest elytral width **(EW)**; head length (**HL**) was measured along the anterior margin of labrum to the posterior margin tumid gena; head width (**HW**) was measured along the widest part of head including eyes; pronotum length (**PL**) was measured along the median line of pronotum; pronotum width (**PW**) was measured across the widest part of the pronotum; elytra length (**EL**) was measured along the suture from base of scutellum to the elytral apex.

Dry specimens were soaked in hot water for 1–2 hours to soften the body. The abdomen was opened at its lateroapical margin and genitalia were pulled out using forceps. Genitalia were soaked in warm 10% KOH for 1 hour, and dyed in Chlorazol Black E. The basal orifice of the aedeagus was injected with 100% ethanol by using a microinjector until the internal sac was fully everted. The aedeagus with its everted internal sac was photographed by a Canon D450 camera fitted to a Nikon SMZ1500 stereomicroscope or a Nikon D610 digital camera fitted to a Nikon SMZ18 stereomicroscope. The photographs were combined with Helicon Focus software to obtain one synthesized photograph, and finally edited in Photoshop (CC). A microvial with genitalia was pinned to the specimen from which the genitalia were removed for storage. Collections cited in this article are indicated by the following abbreviations:

**CBWX**Collection of Bi Wenxuan, Shanghai, China.

**IZCAS** Institute of Zoology, Chinese Academy of Sciences, Beijing, China.

**KIZ**Kunming Institute of Zoology, Chinese Academy of Sciences, Kunming, China.

**NHMUK**The Natural History Museum, London, UKThe Natural History Museum, London, UK.

## Taxonomy

### Genus Manipuria

Taxon classificationAnimaliaColeopteraChrysomelidae

Jacoby, 1908

43B05C47-666A-5B3F-8D4C-215B181BFC79

Manipuria Jacoby, 1908: 84; Monrós 1960: 153.

#### Type species.

*Manipuria dohertyi* Jacoby, 1908: 84 (type locality: Manipur; holotype in NHMUK), by monotypy.

#### Diagnosis.

Body elongate and subcylindrical. Head obviously longer than width; eye small; gena elongate, lateral sides behind eyes almost parallel; vertex smooth in the center, sparsely punctate in lateral area, with a longitudinal groove in the middle; occipit punctate, with a longitudinal groove medially; frontal tubercle glabrous, raised; clypeo-frontal area triangular, area near the anterior margin raised, disc with punctures and setae; labrum transverse, with setae on both apical angles. Antenna filiform, more than half BL. Pronotum wider than head, lateral constriction impunctate; posterior transverse impression distinct. Elytra unicolored or with yellow patches; striae with punctures regularly arranged, punctures weakened posteriorly and disappeared apically. Legs slender, with punctures and pubescence. Two claws asymmetrical. Abdominal sternite with pubescence and punctures.

BL. 9.0–14.0 mm.

#### Distribution.

China (Yunnan, Tibet); India (Manipur).

### Manipuria yuae sp. nov.

Taxon classificationAnimaliaColeopteraChrysomelidae

7C17927C-7F6E-5C23-9AD8-55C3085435E3

http://zoobank.org/1C8831C4-A2E8-484F-B356-B004BEFA86E1

Figures 1–3 , 5–8 , 9–10 , 15–16 , 18–19 , 21 , 22

#### Type locality.

China, Tibet, Mêdog, Baibung, Gelin village, altitude 1171 m.

#### Type material.

***Holotype***: male (IZCAS), China, Tibet, Mêdog, Baibung, Gelin village, 29.40322N, 95.18435E/1171 m, 2011.VIII.13, Wenxuan Bi coll./HOLOTYPE *Manipuria yuae* sp. nov., des. by Xu, Bi & Liang, 2020 [red label]. ***Paratype*** (two males and one female): one male (IZCAS), the same collecting data as holotype but labeled as paratype; one female (IZCAS), China, Tibet, Mêdog, Baibung, Gelin village, 29.40322°N, 95.18435°E/1171 m, 2011.VIII.11, Xiaodong Yang coll./PARATYPE *Manipuria yuae* sp. nov., des. by Xu, Bi & Liang, 2020; one male (KIZ), China, Yunnan, Ruili, 1981.X.16, Dazhi Dong coll./PARATYPE *Manipuria yuae* sp. nov., des. by Xu, Bi & Liang, 2020 [yellow label].

#### Diagnosis.

Body brownish red. Head longer than wide; gena elongate with wrinkles and setae, lateral sides behind eyes almost parallel, slightly constricted behind gena; antennae more than half of BL, 5–11 antennomeres cylindrical. Pronotal disc with fine punctures, lateral sides constricted after the middle. Scutellum triangular. Elytra with punctures regularly arranged, punctures absent apically.

#### Comparisons.

This species can be distinguished from *M. dohertyi* by the following combination of characters: lateral sides of head without prolongation in front of mandible, elytral unicolored (lateral sides of head with a tooth-like prolongation in front of mandible, elytra with four yellow patches in *M. dohertyi*).

#### Description.

BL = 12.4–14.0 mm, BW = 4.2–4.5 mm. Head, pronotum, elytra, antennae, tibia, metasternum, abdominal sternite brownish red; prosternum, mesosternum, metepisternum black; ventral surface of femora brown, dorsal surface black; basal three tarsomeres brown, apical tarsomere black.

**Figures 1–4.**
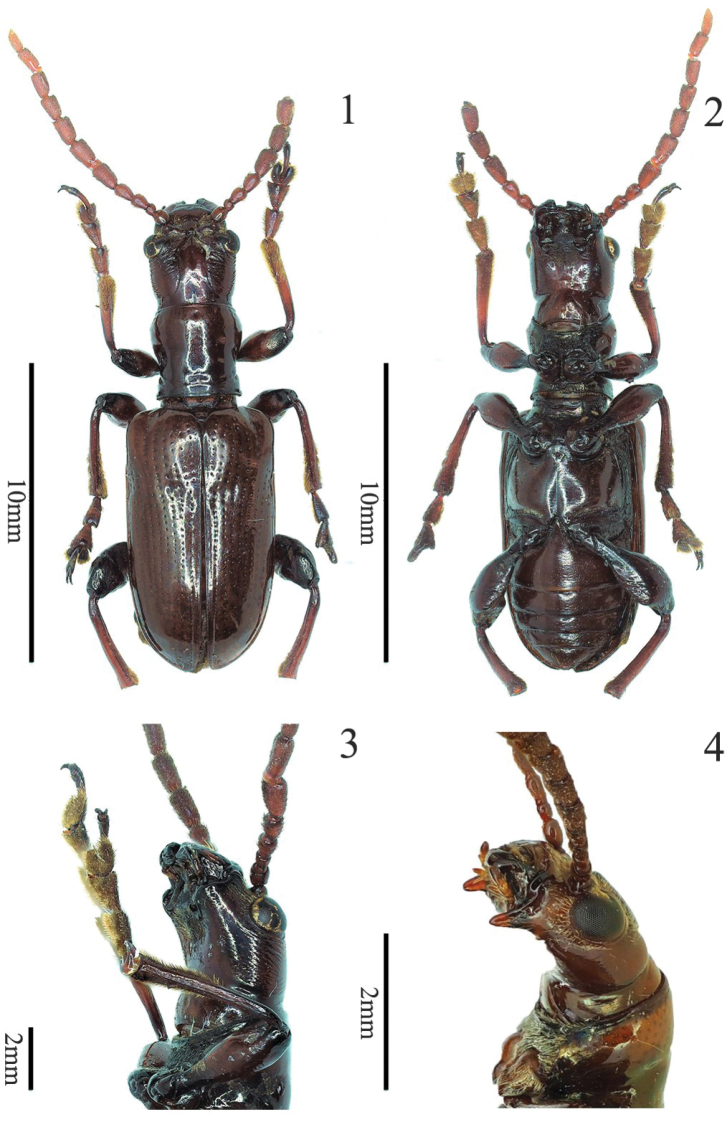
*Manipuria* spp. **1** holotype of *M. yuae* sp. nov., dorsal view **2** holotype of *M. yuae* sp. nov., ventral view **3** head of holotype of *M. yuae* sp. nov., ventro-lateral view **4** head of *M. dohertyi*, male, ventro-lateral view.

**Figures 5–8.**
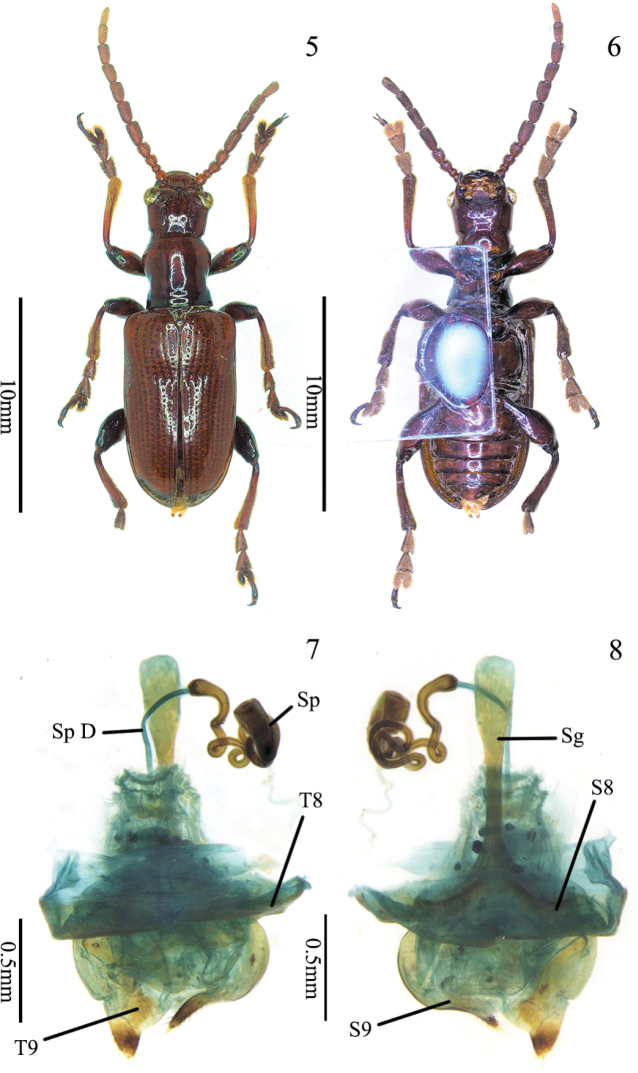
Paratype of *Manipuria yuae* sp. nov., female **5** habitus, dorsal view **6** habitus, ventral view **7** female genitalia and reproductive organs, dorsal view **8** genitalia and reproductive organs, ventral view. Abbreviations: Sg spiculum gastrale; S8 sternite 8; S9 sternite 9; T8 tergite 8; T9 tergite 9; Sp D spermathecal duct; Sp spermatheca.

**Figures 9, 10.**
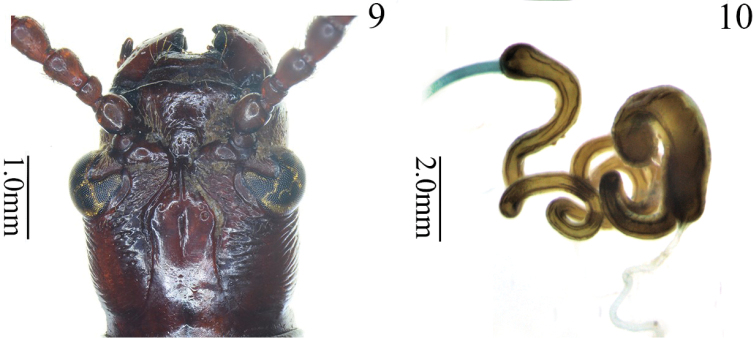
*Manipuria yuae* sp. nov. **9** head, dorsal view **10** spermatheca and spermathecal gland.

***Head*** (Figs 1, 3, 5, 9). HL/HW = 1.5–1.6; gena elongate, lateral sides behind eyes almost parallel, head slightly constricted after gena, area behind eyes with wrinkles and setae; vertex smooth in the center, punctate in lateral area, with a deep groove in the middle, with apex pointed dorsally before groove (Fig. 9); occipit with a shallow furrow medially, sparsely punctate; frontal tubercle glabrous, raised; clypeo-frontal area triangular, area near the anterior margin raised, disc with punctures and setae; labrum transverse, with 3–5 long setae on each outer apical angle; antennae filiform, more than half of BL, antennomeres 1 and 2 nearly globular, shiny, antennomere 1 twice as long as antennomere 2, antennomeres 3 and 4 pubescent and punctate, length almost equal, antennomeres 5–11 cylindrical, with punctures and pubescence, antennomeres 5–7 twice as long as wide, antennomeres 8–11 three times as long as wide.

***Pronotum*.**PW/HW =1.0–1.1, PL/PW = 1.1–1.2; anterior angle protruding, posterior angle not protruding; lateral side constricted behind the middle; areas near anterior and posterior margins with a few fine punctures, middle areas of disc with four rows of irregular fine punctures; posterior transverse impression distinct, basal transverse groove weak. Scutellum triangular, posterior angles round, lateral area of base sparsely pubescent.

***Elytra*** narrowed posteriorly. EL/EW = 1.7; suture angle rounded; humeri protruding, humeral groove shallow, basal impression distinct; striae with punctures regularly arranged, punctures in basal impression larger, remaining punctures smaller, and punctures disappeared apically, intervals with a few fine punctures; scutellar stria composed of 8–11 punctures; epipleura raised, with a row of small punctures; underside of the hind sutural angles with plectrum.

***Mesosternum*** pubescent; mesosternal process short, narrow, densely pubescent, pointed ventrally. metasternal disc with very sparse setae; metepisternum densely pubescent.

***Abdominal*** sternite with sparse setae and punctures, transverse impressions distinct in both lateral areas of each sternite; the eighth visible abdominal tergite with pars stidens.

**Figures 11–14.**
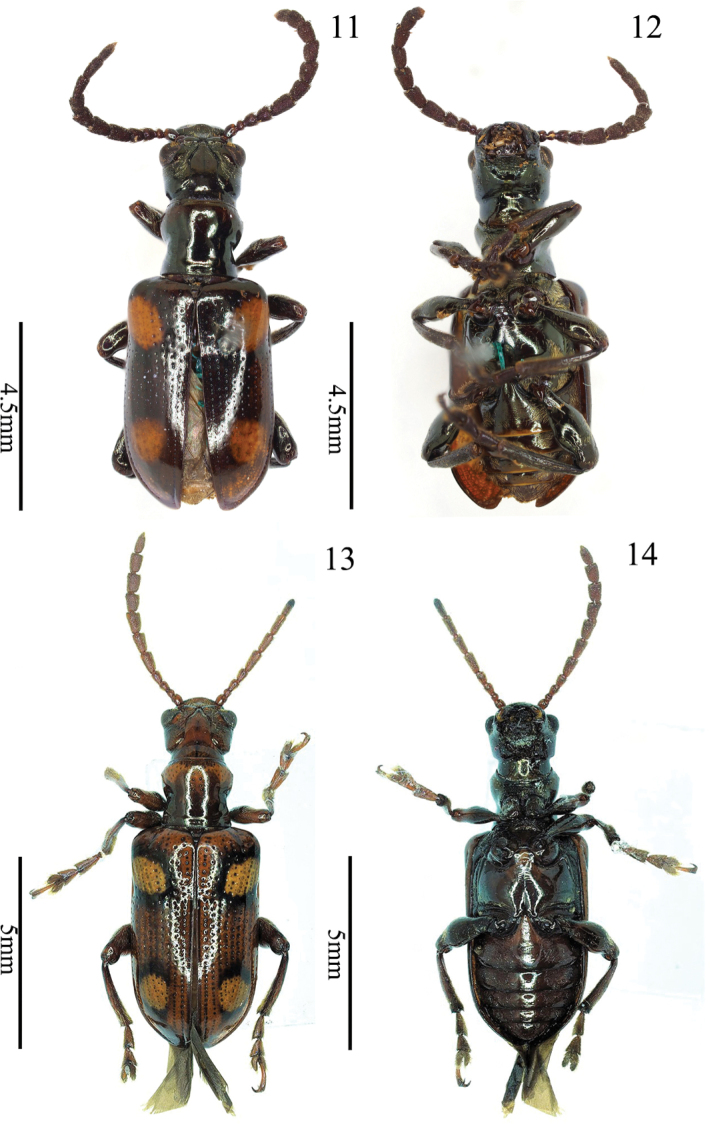
*Manipuria dohertyi* Jacoby **11** type, female, dorsal view **12** type, female, ventral view **13** specimen from Yunnan, male, dorsal view **14** specimen from Yunnan, male, ventral view.

***Legs*** slender; tibia with dense punctures and pubescence; metafemur with dense setae in dorsal surface, with sparse setae in ventral surface, middle area with a large triangular denticle.

**Figures 15–20.**
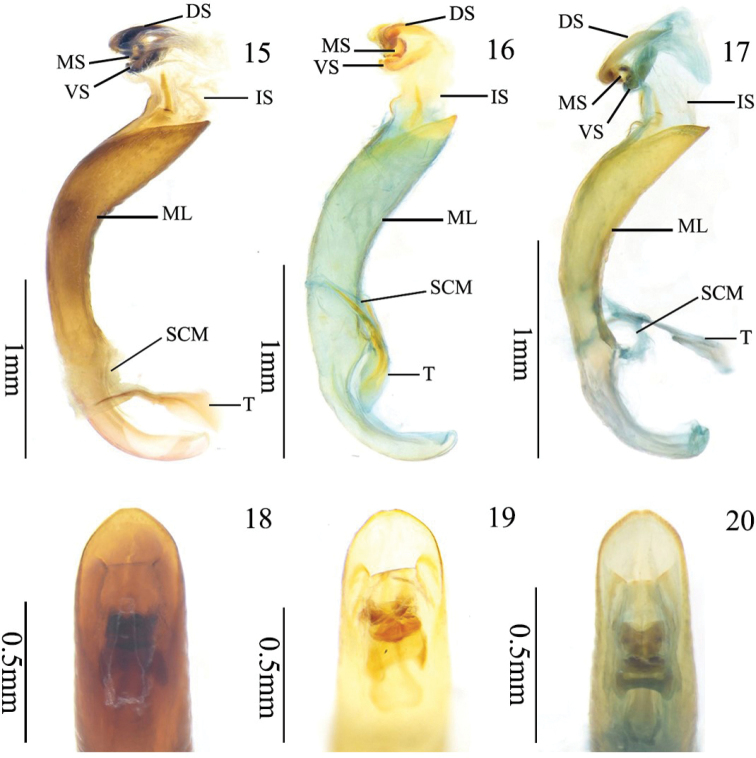
Aedeagus and internal sac of *Manipuria* spp. **15** holotype of *M. yuae* sp. nov., lateral view **16** paratype of *M. yuae* from Yunnan, lateral view **17***M. dohertyi* from Yunnan, lateral view **18** holotype of *M. yuae* sp. nov., dorsal view **19** paratype of *M. yuae* sp. nov. from Yunnan in dorsal view **20***M. dohertyi* from Yunnan, dorsal view. Abbreviations: DS dorsal sclerite; MS dorsal sclerite; VS dorsal sclerite; IS internal sac; ML median lobe; SCM second connecting membrane; T tegmen.

**Figures 21–23.**
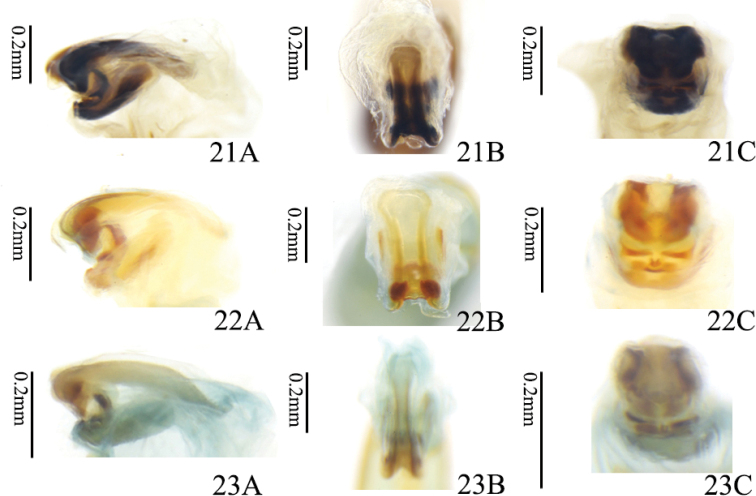
Sclerites in internal sac **21** holotype of *Manipuria yuae***22** paratype of *M. yuae* from Yunnan **23***M. dohertyi* from Yunnan **A** lateral view **B** dorsal view **C** ventral view.

**Male genitalia.** Median lobe strongly sclerotized, tubular, curved, apical portion gradually narrowed, median foramen occupying 1/5 length of aedeagus (Figs 15, 16); apex truncated in the middle (Figs 18, 19); tegmen Y-shaped and slender, basal piece of tegmen triangular and relatively broad, lateral lobes narrow and combined with second connecting membrane; internal sac membranous, with dorsal, median, and ventral strongly scleritized (Figs 21A–C, 22A–C).

**Female reproductive organs** (Figs 7, 8, 10). Tergites 8 and 9, sternites 8 and 9 of female sclerotized, posterior areas of tergite 8 and sternite 8 with sparse setae and apodemes, spiculum gastrale Y-shaped and expanded in the end, distal part of spiculum gastrale squared, margin curved; ovipositor with dense setae, distal part of ovipositor conical, short; spermatheca strongly sclerotized, complicatedly folded and curved; spermathecal duct relatively long, connected with spermatheca and bursa copulatrix; spermathecal gland curved, long.

#### Distribution.

China (Tibet, Yunnan).

#### Etymology.

The specific name *yuae* is proposed in memory of Professor Peiyu Yu, who contributed greatly to the taxonomy on Chinese Criocerinae.

#### Host plant.

This species lived on *Smilax* sp. (Smilacaceae) according to observations of the second author (BWX).

#### Habitat.

The locality of the new species in Mêdog county is situated at the northernmost part of tropics in Asia. The vegetation is tropical seasonal rainforest. The climate is characteristic of high temperature, plentiful precipitation, and high humidity. The biodiversity is rich in this region. The forests are composed of tall trees, woody vines and epiphytes. The host plant of new species is *Smilax* sp. (Smilacaceae). It shares its habitat with plants such as *Hedychium* sp. (Zingiberaceae), *Musa* sp. (Musaceae), *Epipremnum* sp. (Araceae), *Lysionotis* spp. (Gesneriaceae), *Alsophila* sp. (Cyatheaceae), and *Dryopteris* sp. (Dryopteridaceae).

#### Remarks.

The specimen from Yunnan differs slightly from those from Tibet in having a lighter color (yellow-red), a shorter head (HL/HW = 1.5), and weaker sclerites (yellow) of the male genitalia.

When this new species is included, the concept of the genus *Manipuria* is expanded slightly by the absence of a projection on front of the mandible. The genus *Manipuria* differs from the genus *Lilioceris* mainly in having elongate gena, a less constricted neck, and small eyes.

Some characters of the new species suggest its intermediate position between *Lilioceris* and *Manipuria* as the head is only slightly enlarged and the ventral teeth on the head only weakly developed. It appears to occupy a position nearer to *Lilioceris* than to *Crioceris* as the pronotum is strongly narrowed in the middle. The relationship between *Manipuria* and *Lilioceris* needs further investigation.

### Manipuria dohertyi

Taxon classificationAnimaliaColeopteraChrysomelidae

Jacoby, 1908

6B56C337-C539-5CAA-B2DF-1924BB5CA0E5

Figures 4 , 11–14 , 17 , 20 , 23

Manipuria dohertyi Jacoby, 1908: 84; Monrós 1960: 153.

#### Material examined.

**Types.** One female (NHMUK), Type / Doherty / India Or, Manipur / Fry Coll. 1905-100 / Manipuria Dohertyi Jac. / Syntype. **Non-types.** one male (IZCAS), China, Yunnan, Gongshan, Dulongjiang, Maku village, 27.68936°N, 98.30804°E/1692 m, 2019.8.22, Liang HB & Xu Y coll. one female (CBWX), China, Yunnan, Gongshan, Dulongjiang, Maku village / 1250 m, 2015.7.25, Bi WX coll.

#### Diagnosis.

Body brownish black, elytra with yellow patches, each patch surrounded with a black circle. Head longer than wide; lateral sides of head with a tooth-like prolongation in front of mandible; gena elongate, with fine wrinkles and setae; lateral sides behind eyes almost parallel; antenna more than half BL. Pronotal disc with fine punctures; lateral sides constricted behind the middle. Scutellum triangular.

#### Redescription.

BL = 8.7–9.0 mm, BW = 3.0–3.2 mm. Brown or brownish black, with coppery metallic luster, each elytron with two yellow patches, one patch behind the shoulders slightly transverse, and another near the apex rounded, each surrounded by a black circle.

***Head*** (Figs 4, 11, 13). HL/HW = 1.1; lateral sides of head with a tooth-like prolongation in front of mandible, gena elongate with fine wrinkles and setae, lateral sides behind eyes almost parallel, then constricted behind gena; vertex smooth, with a shallow longitudinal groove in the middle, apex pointed dorsally before the groove; occipit sparsely punctate, with a shallow longitudinal groove medially; frontal tubercle glabrous, raised; clypeo-frontal area triangular, area near anterior margin raised, disc with punctures and setae; labrum transverse, with 3–5 long setae on both apical angles; antenna filiform, more than half BL, antennomeres 1 and 2 nearly globular and shiny, antennomere 1 twice as long as antennomere 2, antennomeres 3 and 4 pubescent and punctate, length almost equal, antennomeres 5–11 cylindrical, with punctures and pubescence, antennomeres twice as long as wide.

***Pronotum*.**PW/HW = 1.1–1.3, PL/PW = 0.9–1.0; anterior angle protruding, posterior angle not protruding; lateral side constricted just behind the middle; middle region of disc with two rows of fine punctures and a longitudinal fovea; posterior transverse impression distinct; basal transverse groove weak. Scutellum triangular, lateral sides of base with pubescence.

***Elytra*** narrowed posteriorly, EL/EW =1.7; suture angle rounded; humeri protruding, humeral groove shallow; basal impression distinct; punctures in basal impression large, remaining punctures small, apical punctures disappeared, intervals with few fine punctures; scutellar stria composed of 3–6 punctures; epipleura raised, with a single row of small punctures; underside of the hind sutural angles with plectrum.

***Mesosternum*** pubescent, mesosternal process short, narrow, densely pubescent, pointed ventrally. Outer metasternal disc with an oblique setose area, extending from posterior angle to the middle of disc (setae partially fell off in Fig. 14, but their pores still visible); metepisternum densely pubescent.

***Abdominal*** sternite with dense pubescence and punctures, middle area of sternite less pubescent than both sides, transverse impressions distinct in both lateral areas; the eighth visible abdominal tergite with pars stidens.

***Legs*** slender; tibia with punctures and pubescence; femora with dense setae in dorsal surface, with sparse setae in ventral surface, middle area with a triangular denticle.

***Genitalia*.** Median lobe sclerotized, tubular, curved, median foramen occupying 1/4 length of aedeagus; medial portion slightly broader than basal and apical portion in ventral view (Fig. 17); middle of apex truncated in dorsal view (Fig. 20); tegmen Y-shaped and weak, basal piece of tegmen triangle and relatively small, lateral lobes slender and combined with second connecting membrane; internal sac membranous, with three sclerotized sclerites, dorsal, median, and ventral sclerites (Fig. 23A–C).

#### Distribution.

China (Yunnan), India (Manipur).

#### Host plant.

This species lived on *Smilax ferox* Wall. ex Kunth (Smilacaceae) according to photos (Figs 25, 26) taken by the second author (BWX).

#### Habitat.

**Figure 24.**
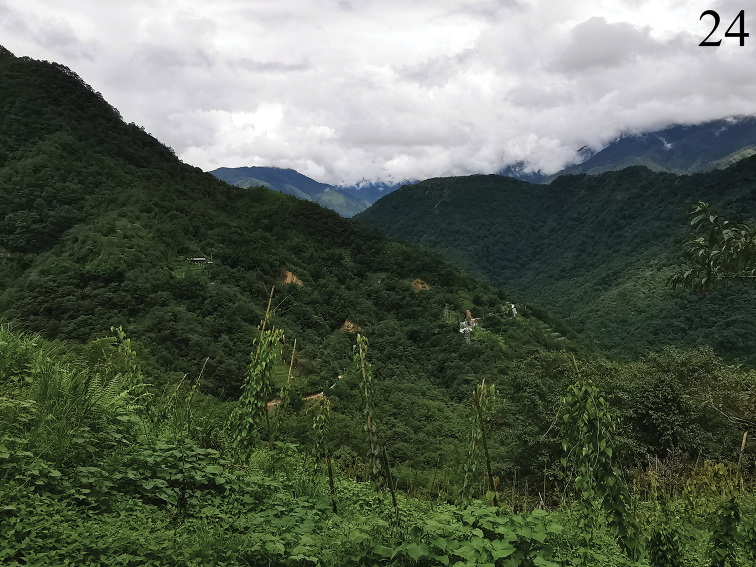
Habitat of *Manipuria dohertyi* in Maku village of Yunnan, China.

The habitats are shown in Fig. 24. It is similar to those of *M. yuae* sp. nov. in Mêdog, but with some patches of cultivated field.

#### Remarks.

The specimens from Yunnan, China differ slightly from the type specimen in having: 1) body color brown (brownish black in type); 2) sides behind eyes and outer area of metasternal disc with sparser setae (denser in type); 3) antennomeres 3 and 4 longer (shorter in types); 4) anterior yellow patches more distant from shoulder and each of four patches surrounded by a distinct blackish circle (anterior yellow patches closer to shoulder and patches surrounded by a weak blackish circle in type). These external variations indicate that the specimens from Yunnan might represent another new species, but we only checked two specimens from Yunnan and tentatively treated them as members of *M. dohertyi*. These new records extend the distribution of *M. dohertyi* northwards by ca 500 km.

We also compared the differences in the internal sac between *M. dohertyi* and *M. yuae*, and they differ significantly in the shape of the dorsal sclerite (Figs 21–23). In lateral view, the dorsal sclerite of *M. dohertyi* is obviously enlarged backwards but *M. yuae* does not have such a sclerite; in dorsal view, the dorsal sclerite of *M. dohertyi* is slender and narrowed in the middle, and the sides of the dorsal sclerite of *M. yuae* are nearly parallel; in ventral view, the horn of the dorsal sclerite of *M. yuae* is bent downwards, but in *M. dohertyi* it is bent forwards.

**Figure 25.**
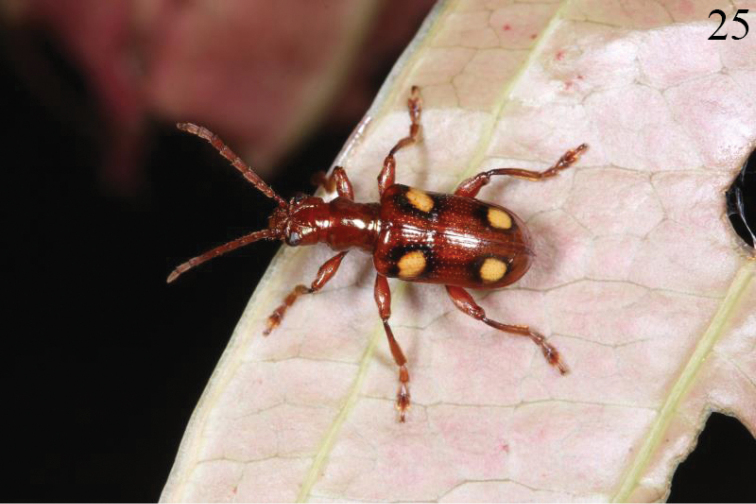
*Manipuria dohertyi* Jacoby standing on leaf of *Smilax*.

**Figure 26.**
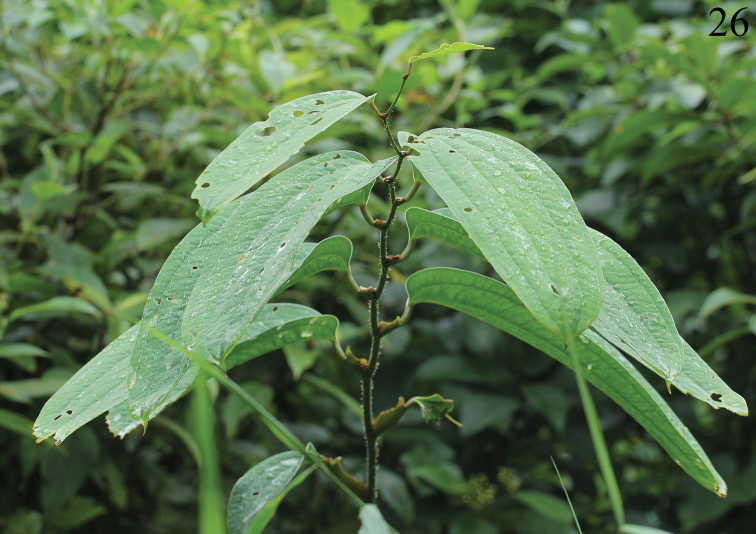
*Smilax ferox* Wall. ex Kunth (host plant of *Manipuria dohertyi*).

## Supplementary Material

XML Treatment for Manipuria

XML Treatment for Manipuria yuae

XML Treatment for Manipuria dohertyi
